# The "Times" on the Medical Profession

**Published:** 1912-11-16

**Authors:** 


### THE "TIMES" ON THE MEDICAL PROFESSION.

In its issue of the 7th inst., and under the title "What is a Quack?" the *Times* published a leading article which can only be described as a deliberate attack on the medical profession. Members of the profession who read the article will readily detect the animus of the writer. But the public can hardly be expected to be equally well informed, and doubtless many persons will read the article and will accept its statements in all good faith. As a model of journalistic enterprise and efficiency the *Times* rightly commands widespread admiration and confidence, and it is, therefore, the more to be regretted that it has lent itself to a proceeding which cannot but be regarded, at least by the medical profession, as unfair and unjust.

The scope of the article here in question is a somewhat extensive one, but its main proposition is that the profession stigmatises as a "quack" any technically unqualified person who attempts to cure disease, and also any one of its own members who "acts in a manner disapproved by the majority dominant at the moment," or who presumes "to prescribe his drugs upon a principle not accepted by others." With the first half of this indictment we do not propose at this moment to concern ourselves. Quacksalvers have been known in all ages, and even the *Times* will hardly deny that some members of the series still exist. There are, no doubt, degrees in quackery, and in the rough classification of practical life possibly some honest, even if mistaken, men have been grouped with the deliberate charlatans. This, in so far as it has occurred, is, we admit, a matter for regret, but we must at the same time plead that distinction between the groups is not easy.

The second head of our offending, however, is in a different position, and, fortunately, the issue is one which can be tested by fact and experience. The charge is a very direct one, and, as will be seen from the words quoted above, it accuses the profession of stigmatising as a "quack" any practitioner who advocates or pursues methods of treatment "disapproved by the majority dominant at the moment." A ready answer to this wild statement can promptly be supplied by anyone who has a knowledge of current medical literature or of the proceedings of our medical societies. In these are to be found repeatedly the presentation of doctrines and methods which are regarded as unfounded or unwise by the great majority of the profession, yet the authors of these doctrines and methods, so far from being condemned as "quacks," receive a free platform and an impartial hearing. Their convictions, even though not generally approved, are respected as those of honest men, and their work is tested in the crucible of debate and experience. It is not desirable to mention individual names, but we venture to quote two instances in refutation of the indictment formulated by the *Times*. Take, in the first place, the causation of leprosy, and, in the second, the influence of uric acid in the production of disease. In connection with each of these a special and particular view is held and announced by a well-known member of the medical profession, a view, too, which is "disapproved by the majority dominant at the moment." Are the authors of these doctrines and of certain methods of treatment which arise out of them condemned as "quacks"? The answer is that these gentlemen enjoy the respect and regard of all their colleagues, and that their teaching receives a full opportunity in the columns of our journals and in the proceedings of our societies. Yet the *Times* has the courage to tell the British public that if any member of the profession presumes "to prescribe his drugs upon a principle not accepted by others . . . he, too, comes under the ban."

One of the illustrations advanced in support of this charge is that if any medical practitioner "says that likes may be cured by likes, instead of opposites by opposites, he is . . . the most hated of all quacks." To this we reply that if any practitioner cares to amuse himself by proclaiming either one or other of these barren propositions he is quite free to do so, though we doubt whether he will attract an audience. But if he is able to produce some agent or method which will certainly act favourably in the treatment of some given disease, whether "like" or "opposite," the whole profession will give him attention. Of course, if he prefers a public audience, and endeavours to attract this by the assumption of a sectarian title and by an open proclamation that he alone possesses the sound and correct method, he cannot be surprised if his *confrères* view him with suspicion; and history affords abundant instances to justify this attitude. Whether such men are "quacks" or not is a question of definition, but the suspicion which attaches itself to them is no justification for random charges of intolerance, bigotry, and malevolence.

The writer of the *Times* article is good enough to advise the profession how to behave. When he has gained some knowledge of the true motives of the profession and has freed his mind from bias and ill-will he may perhaps presume to play the censor. In the meantime, he seems to us no better qualified for his self-appointed task than we ourselves should be to advise on the methods of selling an encyclopædia or booming a circulating library.

